# Decoding the causes of ischemic stroke despite ongoing oral anticoagulation and their clinical impact: the ASPERA-R study

**DOI:** 10.1007/s00415-026-14039-x

**Published:** 2026-08-17

**Authors:** Federico De Santis, Matteo Foschi, Francesca Gabriele, Lucio D’Anna, Andrea Zini, Matteo Paolucci, Stefano Forlivesi, Ludovica Migliaccio, Maria Maddalena Viola, Angelo Cascio Rizzo, Maria Sessa, Ghil Schwarz, Rachele Tortorella, Soma Banerjee, Gaurav Desai, Kazi Farhad Ahmed, Gabriele Prandin, Leonardo Pantoni, Francesco Mele, Giuseppe Scopelliti, Ilaria Cova, Mariarosaria Valente, Domenico Maisano, Luca Antonelli, Maria Rosaria Bagnato, Giovanni Di Mauro, Francesca Bernocchi, Martina Gaia Di Donna, Barbara Casolla, Souleymane-M.-Bara Diallo, Baptiste Alvarez, Julie Contenti, Laura González-Martìn, Ricardo Rigual, Blanca Fuentes, Carlos Hervás-Testal, Paolo Candelaresi, Vincenzo Andreone, Antonio De Mase, Emanuele Spina, Diana Aguiar de Sousa, Mariana Almudi Souza, Alberto Fior, Miguel Serôdio, Pietro Caliandro, Aurelia Zauli, Giuseppe Reale, Ahmed Abdelalim, Sandra Ahmed, Samah Ali Ismail, Liqun Zhang, Tara Latimer, Muhammad Elboghdady, Ahmed Elbassiouny, Tamer Roushdy, Hossam Shokri, Federica Ferrari, Nicola Davide Loizzo, Federico Mazzacane, Maria Guarino, Valentina Barone, Paola Forti, Giuseppe Rinaldi, Marco Vito Rossi, Vincenzo Laterza, Giovanni Frisullo, Pier Andrea Rizzo, Aldobrando Broccolini, Marina Mannino, Valeria Terruso, Marcella Caggiula, Annalisa Rizzo, Ana Catarina Fonseca, Bernardo Antunes, Ana M. Barbosa, Hrvoje Budincevic, Petra Crnac, Giovanna Viticchi, Mauro Silvestrini, Lorenzo Barba, Viktoria Musienko, Markus Otto, Piergiorgio Lochner, Benjamin Landau, Sandeep Buddha, Roumeisa Khalil, Maria Grazia Piscaglia, Elena Minguzzi, Marialuisa Zedde, Ahmed Nasreldein, Luisa Vinciguerra, Luis Rufo Costa, Ahmed Elsaid Elsayed, Mona AlBanna, Laura Tudisco, Maria Giulia Mosconi, Alexandros A. Polymeris, Giovanni Merlino, Simona Sacco, Raffaele Ornello

**Affiliations:** 1 Department of Biotechnological and Applied Clinical Sciences, University of L’Aquila, Via Vetoio Snc, 67100 L’Aquila, Italy; 2 Department of Neurosciences, Stroke Unit – Neurology Unit, S.Maria Delle Croci Hospital, AUSL Romagna, Ravenna, Italy; 3 Department of Stroke and Neuroscience, Charing Cross Hospital, Imperial College London NHS Healthcare Trust, London, UK; 4 Department of Brain Sciences, Imperial College London, London, UK; 5 Department of Neurology and Stroke Center, IRCCS Istituto Delle Scienze Neurologiche Di Bologna, Maggiore Hospital, Bologna, Italy; 6 Department of Neurology and Stroke Unit, ASST Grande Ospedale Metropolitano Niguarda, Milan, Italy; 7 Clinical Unit of Neurology, Department of Medicine, Surgery and Health Sciences, University Hospital and Health Services of Trieste, ASUGI, University of Trieste, Trieste, Italy; 8 Neuroscience Research Center, Department of Biomedical and Clinical Sciences, University of Milan, Milan, Italy; 9 Department of Neurorehabilitation Sciences, Casa Di Cura Igea, Milan, Italy; 10 Neurology Unit, University Hospital Luigi Sacco, Milan, Italy; 11 Clinical Neurology, DMED, University of Udine, Udine, Italy; 12 UOC Stroke Unit E Neurologia, Ospedale Fabrizio Spaziani, Frosinone, Italy; 13 Stroke Unit, CHU Pasteur 2, Université Cote d’Azur, UMR2CA URRIS, Nice, France; 14 Emergency Department, Pasteur 2 Hospital, University Hospital Center of Nice, Nice, France; 15 Stroke Center and Department of Neurology, Hospital La Paz Institute for Health Research-IdiPAZ (La Paz University Hospital-Universidad Autónoma de Madrid), Madrid, Spain; 16 UOC Neurologia E Stroke Unit, AORN Cardarelli, Naples, Italy; 17 Stroke Center, Department of Neurosciences, Centro Hospitalar Universitário Lisboa Central - ULS São José, Lisbon, Portugal; 18 Faculdade de Medicina, Universidade de Lisboa, Lisbon, Portugal; 19 Department of Neuroscience, Catholic University of the Sacred Hearth, Rome, Italy; 20 UOC Neurology, Department of Neuroscience, Sensory Organs, and Thorax, Fondazione Policlinico Universitario A. Gemelli IRCCS, Rome, Italy; 21 UOC Neuroriabilitazione Ad Alta Intensità, Fondazione Policlinico Universitario A. Gemelli IRCCS, Rome, Italy; 22 Cairo University Stroke Center, Department of Neurology, Faculty of Medicine, Cairo University, Giza, Egypt; 23 Department of Neurology, St George’s University Hospital, London, UK; 24 Neurology Department, Faculty of Medicine, Ain Shams University, Cairo, Egypt; 25 Department of Brain and Behavioral Sciences, University of Pavia, Pavia, Italy; 26 Department of Emergency Neurology and Stroke Unit, IRCCS Mondino Foundation, Pavia, Italy; 27 IRCCS Istituto Delle Scienze Neurologiche Di Bologna, Bologna, Italy; 28 Department of Medical and Surgical Sciences, University of Bologna, Bologna, Italy; 29 IRCCS Azienda Ospedaliero-Universitaria Di Bologna, Bologna, Italy; 30 S.C. Neurologia, Ospedale “Di Venere”, Bari, Italy; 31 Emergency Neurology - Department of Neuroscience, Sensory Organs, and Thorax - Policlinico Universitario Agostino Gemelli, IRCCS Rome, Rome, Italy; 32 Catholic University of the Sacred Heart - Fondazione Policlinico Agostino Gemelli IRCCS, Rome, Italy; 33 Stroke Unit - Department of Neuroscience, Sensory Organs, and Thorax - Policlinico Universitario Agostino Gemelli, IRCCS Rome, Rome, Italy; 34 UOC Neurologia E Stroke Unit, AOOR Villa Sofia-Cervello, Palermo, Italy; 35 UOC Neurologia E Stroke Unit, PO Vito Fazzi, Lecce, Italy; 36 Neurology Department, Hospital de Santa Maria, Faculdade de Medicina, University of Lisbon, Lisbon, Portugal; 37 Department of Neurology, Hospital de Santa Maria, Lisbon, Portugal; 38 Department of Neurology, Sveti Duh University Hospital, Zagreb, Croatia; 39 Department of Neurology and Neurosurgery, Faculty of Medicine, J.J, Strossmayer University of Osijek, Osijek, Croatia; 40 Department of Psychiatry and Neurology, Faculty of Dental Medicine and Health, J.J. Strossmayer, University of Osijek, Osijek, Croatia; 41 Neurological Clinic, Marche Polytechnic University, Ancona, Italy; 42 Department of Neurology, Martin-Luther-University Halle-Wittenberg, Halle, Germany; 43 Department of Neurology, Saarland University Medical Center, 66421 Homburg, Germany; 44 Department of Stroke Medicine, Southmead Hospital, North Bristol NHS Trust, Bristol, UK; 45 Neurology Unit, Stroke Unit, Azienda Unità Sanitaria Locale-IRCCS Di Reggio Emilia, Reggio Emilia, Italy; 46 Department of Neurology, Assiut University, Assiut, Egypt; 47 Department of Neurology and Stroke Unit, ASST Crema Hospital, Crema, Italy; 48 Department of Neurology, Local Health Unit of Alto Minho, Viana Do Castelo, Portugal; 49 Neurology and Neurointervention Department, Kobry Elkoba Medical Complex, Cairo, Egypt; 50 Department of Neurology, National Neuroscience Institute, King Fahad Medical City, Riyadh, Saudi Arabia; 51 Stroke Unit, Careggi University Hospital, Florence, Italy; 52 Department of Internal and Cardiovascular Medicine, Santa Maria Della Misericordia Hospital, Perugia, Italy; 53 Stroke Division, Department of Neurology, Beth Israel Deaconess Medical Center, Harvard Medical School, Boston, MA USA; 54 Department of Neurology and Stroke Center, University Hospital Basel and University of Basel, Basel, Switzerland; 55 SOSD Stroke Unit, Udine University Hospital, Udine, Italy

**Keywords:** Breakthrough ischemic stroke, Oral anticoagulants, Atrial fibrillation, Cancer, Polypharmacy

## Abstract

**Background:**

Oral anticoagulants (OACs), including vitamin K antagonists and direct OACs, reduce stroke risk in atrial fibrillation (AF), yet breakthrough ischemic stroke still occurs in 1–2% of patients annually despite adequate therapy. The mechanisms underlying these events remain unclear. We aimed to identify potential causes of breakthrough ischemic stroke and assess their impact on outcomes, with a focus on drug interactions.

**Methods:**

ASPERA-R is a multicenter retrospective study including patients with ischemic stroke despite ongoing OAC therapy for AF (February 2020–February 2025). Ongoing treatment was defined by DOAC last intake within 48 h or therapeutic INR levels for VKAs. Potential causes included interacting drugs, active cancer, and competing etiologies. Ninety-day outcomes were compared between patients with and without ≥ 1 potential cause using adjusted Cox regression. Inverse probability-weighted analyses (IPW) evaluated the impact of interacting drugs.

**Results:**

Among 1649 patients (median age 80.4 years [IQR 73.2–85.6]; 860 [52.2%] female), most were on DOACs (1275; 77.3%), and 724 (43.9%) had ≥ 1 potential cause. Interacting drugs were identified in 28.4%, competing etiologies in 24.3%, and cancer in 4.4% cases. Patients with one or more potential cause had a higher risk of recurrent ischemic stroke at 90 days (HR 2.04, 95% CI 1.18–3.53) compared with those without, with no differences in other outcomes. In the IPW analyses, interacting drugs were associated with increased myocardial infarction risk (HR 3.26, 95% CI 1.30–8.17).

**Conclusions:**

Potential causes are identifiable in about half of breakthrough strokes and are associated with higher risk of recurrence compared with those without. Improved detection and management of these factors may reduce recurrence risk, warranting individualized approaches.

**Supplementary Information:**

The online version contains supplementary material available at https://doi.org/10.1007/s00415-026-14039-x.

## Introduction

Atrial fibrillation (AF) is associated with a fivefold risk for ischemic stroke compared with no AF because of the risk for cardiac embolism [1]. Oral anticoagulants (OACs)—including vitamin K antagonists (VKAs) and direct OACs (DOACs)—are the mainstay for ischemic stroke prevention in patients with AF [2, 3]. Those drugs led to a substantial reduction in the risk of recurrent ischemic strokes in this population [4–7].

Despite the substantial reduction in the risk of ischemic events in patients with AF, OAC use does not abolish this risk, which is estimated at 8.9% after 1 year in the overall population of patients [8] and even higher in patients with high CHA2DS2-VASc score [9]. Low adherence [10], temporary interruption because of surgical procedures, or inappropriate dosing [11] are established causes of breakthrough ischemic stroke. However, breakthrough ischemic stroke can occur even in patients with optimal adherence and dosing of OACs, due to factors related to the patient (renal or hepatic dysfunction, obesity, older age, malabsorption syndromes, food interactions), to the drug (subtherapeutic dosing, drug–drug interactions, DOAC-specific limitations such as fixed dosing, absorption issues, and lack of monitoring), and or to the disease (concomitant atherosclerosis, hypercoagulable states, cancer-associated thrombosis, persistent intracardiac thrombus, or chronic inflammatory conditions) [12]. Drugs with potential interactions with OACs are particularly relevant as they constitute an easily modifiable risk factor for breakthrough ischemic stroke.

A comprehensive evaluation of factors contributing to ischemic stroke in patients adherent to OAC is essential to improve prevention strategies in patients with AF. Identifying modifiable determinants may enable both upstream interventions to reduce incident events and more targeted management after the index stroke to lower the risk of recurrence and adverse outcomes. In addition, observational data defining these factors, their prevalence, and associated risks are crucial to inform the design of future interventional trials and to ensure appropriate patient selection and interventions.

In the present study, we aimed to describe the potential causes of OAC failure in patients who were adherent to oral anticoagulation at the time of breakthrough ischemic stroke and to evaluate their impact on clinical outcomes, with a specific focus on drug interaction with OACs.

## Methods

Advancing knowledge in ischemic Stroke PatiEnts on oRal Anticoagulants (ASPERA) is a large, multicenter, observational, ambispective real-world study coordinated by the University of L’Aquila. It was developed according to two distinct temporal trajectories with specific aims and dedicated analyses: ASPERA-R (retrospective) and ASPERA-P (prospective). The present study reports results from the ASPERA-R cohort, based on data collected from 35 stroke centers across nine European and North African countries (Appendix 1); the ASPERA-P arm is still ongoing. To report our data, we followed the Strengthening the Reporting of Observational Studies in Epidemiology (STROBE) guidelines [13].

### Ethical procedures

The ASPERA study protocol has been registered and approved by the Ethics Committee of the Abruzzo Region (CETRA) with protocol number 033054/25; it is publicly accessible on the ClinicalTrials.gov database with code NCT06823466. For ASPERA-R, written informed consent was obtained in accordance with the Declaration of Helsinki in countries where consent is required; for deceased or unreachable patients, consent from a legally authorized representative was sought when mandated by local regulations, otherwise a waiver of consent was granted by the relevant ethics committees.

### Study population

Investigators retrieved data from consecutive adult patients who had AF, were taking OACs, and had a breakthrough ischemic stroke between February 2020 and February 2025. To limit selection bias, all consecutive hospital admissions and emergency department evaluations during the study period were screened, and all eligible patients were included. We followed a clinical diagnosis of ischemic stroke according to the World Health Organization (WHO) definition [14] accompanied by the availability of at least one neuroimaging examination (either a non-contrast computed tomography or magnetic resonance imaging of the brain) demonstrating one or more ischemic lesions consistent with patient symptoms.

To be included in the study, potentially eligible patients had to be on a continuous intake of OACs at the time of the index ischemic stroke, defined as uninterrupted intake during the 7 days preceding the index stroke with the last intake within 48 h prior to stroke symptom onset for patients on DOACs, or an international normalized ratio (INR) of ≥ 1.5 in patients on VKAs, regardless of the time elapsed between the last intake and stroke symptom onset. We excluded patients with inadequate DOAC dosing, as defined by any regimen not meeting guideline criteria based on renal function, age, body weight, or drug interactions (including underdosing or unjustified dose reductions). We also excluded patients with poor compliance to OACs, as the aim was to focus on a population with breakthrough ischemic stroke not explained by OAC adherence issues.

### Definition of potential causes of breakthrough ischemic stroke

Potential causes of breakthrough ischemic stroke were selected from those known in the literature [11] and available in the dataset of ASPERA-R. We included among potential causes of failures: 1. competing etiologies, defined as any cause of ischemic stroke different from cardiac embolism according to the Trial of ORG 10172 in Acute Stroke Treatment (TOAST) classification; 2. drugs with potential interactions with OACs, including cytochrome P inducers (antibiotics such as nafcillin, dicloxacillin, rifampin, or rifabutin; antiseizure drugs such as carbamazepine, phenytoin, or phenobarbital; chemotherapy drugs such as apalutamide, enzalutamide, or mitotane) or inhibitors (mexiletine, ciprofloxacin, fluvoxamine, sulfamethoxazole, amiodarone, fluvastatin, ceritinib, cobicistat, ritonavir, clarithromycin, dronedarone, erythromycin, ketoconazole, or voriconazole) [15] or proton pump inhibitors [16, 17]; 3. active cancer, defined as (a) a diagnosis of cancer that occurred within 6 months of the index event or during hospitalization, (b) cancer treatment with radiotherapy, chemotherapy, or surgery or a combination of them within 6 months of the index event, or (c) a previous history of malignancy and a diagnosis of recurrence or metastasis within 6 months of the index event [18]. Other potential causes of breakthrough ischemic stroke, including renal or hepatic dysfunction (based upon quantitative laboratory findings), obesity, malabsorption syndromes, hypercoagulable states different from active cancer, or chronic inflammatory conditions [12], were not available in the ASPERA-R database and therefore were not considered for the present analyses.

### Study variables and procedures

We collected demographical data, clinical characteristics, ongoing therapies on admission, risk factors, neuroimaging findings, laboratory findings, hyperacute treatment, discharge therapies, days of hospitalization, and functional status at baseline and 90 days post-stroke. We also collected 90-day outcome events of breakthrough ischemic stroke. The 90-day period was chosen to maximize the available follow-up data and avoid the biases related to long-term follow-up in retrospective studies. All outcomes were adjudicated by local investigators through a specific electronic follow-up case report form. A comprehensive list of both mandatory and optional baseline variables is reported in Appendix 2.

Data were extracted from hospital records and collected via a standardized Research Electronic Data Capture (REDCap) case report form (CRF) to ensure consistency and accuracy. To maintain data quality, the ASPERA-R electronic database was subject to weekly checks and central monitoring procedures. The final dataset for this analysis was locked on September 1st, 2025.

For the purposes of this analysis, we included only patients for whom 90-day follow-up outcomes were available (see Supplementary Fig. 1). Whenever follow-up information was not documented in the medical records, it was systematically obtained through direct patient contact, thereby ensuring complete outcome ascertainment. Moreover, to minimize potential sources of bias and allow for reliable comparisons, we restricted the cohort to patients without missing information in any of the predefined mandatory baseline variables. The full list of both mandatory and optional baseline variables is provided in Appendix S2.

### Outcomes

The primary outcome of the ASPERA-R study was the 90-day occurrence of a new ischemic stroke, defined as a new neurological deficit with a corresponding acute ischemic lesion at brain neuroimaging, including those with symptom resolution within 24 h. This definition was introduced to clearly distinguish new events from the progression of the index event. Secondary outcomes included the 90-day ordinal modified Rankin Scale (mRS) score shift, 90-day myocardial infarction, all-cause death, and vascular death, defined as any death due to vascular causes (fatal ischemic or hemorrhagic stroke, sudden cardiac death, myocardial infarction, or other vascular events). Safety outcomes included 90-day moderate-to-severe bleeding, 90-day intracranial hemorrhage (ICH), 24-h hemorrhagic transformation (HT), and 24-h symptomatic ICH. However, for the present analyses, we only considered the 90-day outcomes and excluded 24-h outcomes to focus on time-to-event analyses. Bleeding severity was classified according to the Global Utilization of Streptokinase and Tissue Plasminogen Activator for Occluded Coronary Artery (GUSTO) trial classification [19]. All outcomes were ascertained by local investigators and follow-up data were collected through review of 90-day follow-up visits (in-person or structured telephone interviews).

### Statistical plan

We reported categorical variables as absolute numbers with percentages (%) and continuous variables as median and interquartile range (IQR), assuming a non-normal distribution for continuous variables. We reported 90-day outcomes as numbers and proportions with 95% confidence intervals (CIs). Kaplan–Meier curves were also used to report the risk for 90-day outcomes.

Together with the outcomes in the overall population, we reported the difference in outcomes between patients with and without potential causes of breakthrough ischemic stroke. Baseline differences between patients with and without potential causes were computed via the Chi-squared test or Wilcoxon test as appropriate.

All outcomes were reported for the overall cohort and for the two subgroups of patients with and without potential causes of breakthrough ischemic stroke. The risks for 90-day new ischemic stroke, myocardial infarction, all-cause and vascular death, moderate-to-severe bleeding, and ICH were compared between the two subgroups via Cox regression analyses adjusted for age, sex, and vascular risk factors (arterial hypertension, diabetes mellitus, smoking, dyslipidemia, prior myocardial infarction, prior stroke or TIA, prior ICH, peripheral artery disease); we reported hazard ratios (HRs) with 95% confidence intervals (CIs). Kaplan–Meier curves reported cumulative incidence of events, with subgroup differences tested by log rank.

Together with comparisons between patients with and without potential causes of breakthrough ischemic stroke, we focused on the individual impact of drugs interacting with OACs on the outcomes of breakthrough events. The individual impacts of competing ischemic stroke etiologies and of active cancer on the outcomes of breakthrough ischemic stroke have been reported in previous works on the ASPERA-R database [20–22].

To reduce baseline imbalances between patients with and without drugs interacting with OACs, we applied inverse probability weighting (IPW). IPW produces a “pseudo-population” by weighing the individuals of a study population against a propensity score to control for confounding in a robust fashion; however, given the creation of a pseudo-population, numbers and proportions of outcomes vary with respect to the original population. In our study, propensity scores were estimated via logistic regression including age, ethnicity, enrolling center, vascular risk factors, AF type, baseline NIHSS and mRS, and acute reperfusion therapies as covariates. Stabilized inverse weights were assigned to limit the influence of extreme values. Time-to-event outcomes (90-day new ischemic stroke, myocardial infarction, all-cause and vascular death, moderate-to-severe bleeding, and ICH) were analyzed with Cox regression, while the 90-day mRS shift was analyzed using ordinal logistic regression.

All statistical analyses were performed using R software, version 4.2.

## Results

We included 1649 patients with a median age of 80.4 years (IQR 73.2–85.6); 860 patients (52.2%) were female and 1303 (79.0%) were Caucasian. The study flowchart with the number of excluded patients is displayed in Supplementary Fig. 1. At the time of ischemic stroke, 1275 (77.3%) patients were on treatment with DOACs and 374 (22.7%) with VKAs.

### Potential causes of OAC failure

At the onset of the index event, 469 patients (28.4%) were taking concomitant drugs with known interactions with OACs, while a competing etiology other than cardioembolism was recognized in 401 (24.3%) and active cancer was present in 72 (4.4%). Drugs with known interactions with OACs included proton pump inhibitors (450; 27.3%), antiseizure medications (31; 1.9%), H2 inhibitors (3; 0.2%), dexamethasone (3; 0.2%), and ketoconazole (1; 0.1%); details on drugs interacting with OACs are reported in Supplementary Table 1. Notably, all interacting drugs potentially decrease the efficacy of both VKAs and DOACs by inducing cytochrome P. Competing etiologies other than cardioembolism included intra- or extracranial large artery atherosclerosis (240; 14.6%), lacunar stroke (70; 4.2%), other determined etiology (60; 3.6%), and more than one probable etiology (31; 1.9%). Locations of active cancers were gastrointestinal in 10 patients (0.8%), pulmonary in 14 (0.8%), genitourinary in 17 (1.0%), breast in 12 (0.7%), hematological in 14 (0.8%), skin in 1 (0.1%), and other in 4 patients (0.2%). Overall, 724 patients (43.9%) had at least one potential cause of breakthrough ischemic stroke, while 925 (56.1%) had no potential cause; 544 (33.0%) had just one potential cause, while 180 (10.9%) had more than one.

Compared with patients without potential causes of breakthrough ischemic stroke, those with at least one potential cause had a different distribution of ethnicities (*p* = 0.003) and of AF types (*p* < 0.001), a higher proportion of arterial hypertension (87.3% vs 76.3%; *p* < 0.001), dyslipidemia (58.1% vs 45.8%; *p* < 0.001), diabetes mellitus (30.1% vs 24.3%; *p* = 0.009), prior myocardial infarction (29.3% vs 16.6%; *p* < 0.001), prior stroke or TIA (28.7% vs 21.8%, *p* = 0.001), chronic heart failure (21.1% vs 13.4%; *p* < 0.001), chronic kidney failure (20.4% vs 13.0%; *p* < 0.001), peripheral artery disease (7.3% vs 2.9%; *p* < 0.001), and pacemaker positioning (14.9% vs 11.1%, *p* = 0.023; Table 1).

**Table 1:** Comparison of the baseline characteristics of patients with at least one potential cause for breakthrough ischemic stroke vs those without

	No potential cause (*n* = 925, 56.1%)	≥ 1 potential cause (*n* = 724, 43.9%)	*p*-value
Age (median-[IQR])	80.5 (73.3 − 85.6)	80.1 (73.0 − 85.3)	0.769
Female, n (%)	489 (52.9)	371 (51.2)	0.513
Ethnicity, n (%)			**0.003**
Caucasian	718 (77.6)	585 (80.8)	
Hispanic White	52 (5.6)	61 (8.4)	
Black	17 (1.8)	7 (1.0)	
Asian	5 (0.5)	4 (0.6)	
Other	133 (14.4)	67 (9.3)	
Hospitalized			0.847
Stroke unit	842 (94.5)	662 (93.9)	
Intensive care unit	23 (2.6)	19 (2.7)	
Other hospital unit	26 (2.9)	24 (3.4)	
Risk factors (n, %):			
Smoking	89 (9.6)	65 (9.0)	0.656
Hypertension	706 (76.3)	632 (87.3)	** < 0.001**
Dyslipidemia	424 (45.8)	421 (58.1)	** < 0.001**
Diabetes	225 (24.3)	218 (30.1)	**0.009**
Prior myocardial infarction	154 (16.6)	212 (29.3)	** < 0.001**
Prior stroke/TIA	202 (21.8)	208 (28.7)	**0.001**
Prior ICH	13 (1.4)	14 (1.9)	0.402
Chronic heart failure	124 (13.4)	153 (21.1)	** < 0.001**
Chronic liver failure	2 (0.2)	5 (0.7)	0.141
Chronic kidney failure	120 (13.0)	148 (20.4)	** < 0.001**
Peripheral artery disease	27 (2.9)	53 (7.3)	** < 0.001**
Valvular heart disease			0.217
Mitral stenosis	19 (2.1)	14 (1.9)	
Aortic stenosis	11 (1.2)	20 (2.8)	
Mitral insufficiency	24 (2.6)	22 (3.0)	
Aortic insufficiency	5 (0.5)	2 (0.3)	
Multiple	7 (0.8)	8 (1.1)	
Mechanical valve replacement	46 (5.0)	48 (6.6)	0.150
Biological valve replacement	38 (4.1)	43 (5.9)	0.088
Pacemaker	103 (11.1)	108 (14.9)	**0.023**
Atrial fibrillation type			** < 0.001**
Paroxysmal	158 (17.1)	177 (24.4)	
Persistent	129 (13.9)	71 (9.8)	
Long-standing persistent	43(4.6)	33 (4.6)	
Permanent	454 (49.1)	393 (54.3)	
Not classified	141 (15.2)	50 (6.9)	
Anticoagulation type			0.704
VKA	213 (23.1)	161 (22.2)	
DOAC	712 (77.0)	563 (77.8)	
Minor stroke	247 (26.7)	245 (33.8)	**0.002**
Minor-to-moderate stroke	430 (46.5)	371 (51.2)	0.055
NIHSS baseline (median)	12 (5–18)	10 (4–17)	**0.003**
Large vessel occlusion	533 (57.6)	379 (52.3)	**0.004**
IVT	81 (8.8)	58 (8.0)	0.589
EVT	436 (47.1)	296 (40.9)	**0.011**

*DOAC* indicates direct oral anticoagulants; *EVT* endovascular treatment; *ICH* intracerebral hemorrhage; *IQR* interquartile range; *IVT* intravenous thrombolysis; *TIA* transient ischemic attack; *VKA* vitamin K antagonist

Referring to clinical presentation, patients with at least one potential cause of breakthrough ischemic stroke had a higher proportion of minor stroke (NIHSS ≤ 5) presentation (33.8% vs 26.7%, *p* = 0.002) with lower median NIHSS (10 [IQR 5–18] vs 12 [IQR 4–17] and a lower prevalence of large vessel occlusion (52.3% vs 57.6%; *p* = 0.004) compared with patients without potential causes of OAC failure (Table 1).

Supplementary Table 2 compares the baseline characteristics among patients with one, multiple, or no potential causes of breakthrough ischemic stroke. Patients with either one or multiple potential causes of breakthrough ischemic stroke differed similarly from those without any potential causes. In contrast, there were no significant differences between patients with one versus multiple potential causes of breakthrough ischemic stroke (Supplementary Table 2).

**Fig. 1:**
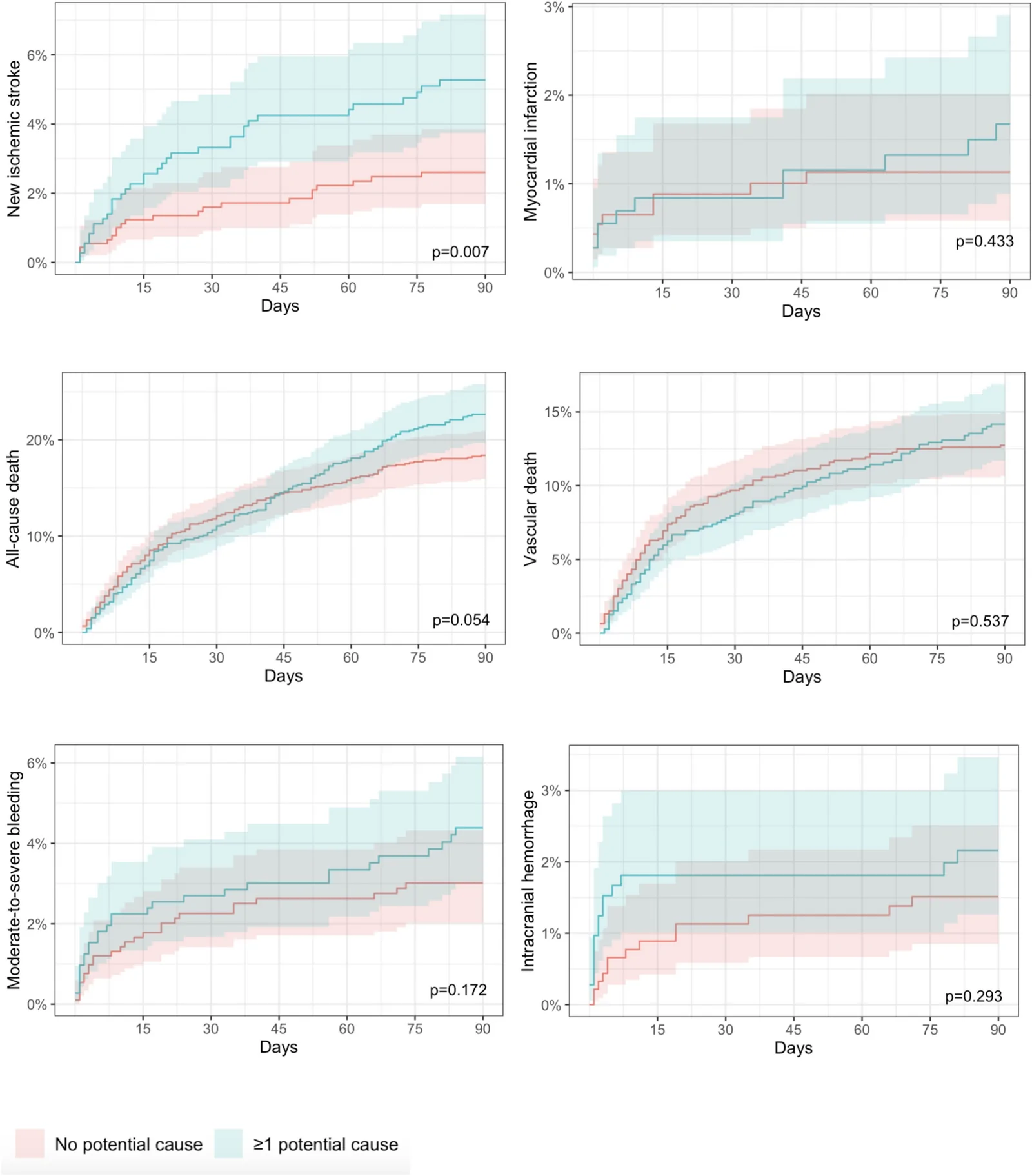
Log-rank analysis of 90-day outcomes showing the comparison between patients with and without potential causes for breakthrough ischemic stroke. P values are for log-rank test

The median (IQR) time to OAC restart was 7 (3–12) days. At the 90-day follow-up, 57 patients (3.5%) had a new ischemic stroke, while 21 (1.3%) had a myocardial infarction; 334 patients (20.3%) died, 215 of them (64.4%) because of vascular causes; 28 patients (1.7%) had an intracranial hemorrhage and 55 (3.3%) moderate-to-severe bleeding events (Table 2). Supplementary Fig. 2 shows details on outcome events according to OAC status (ongoing vs not ongoing). Patients with at least one potential cause of breakthrough ischemic stroke had a significantly higher risk of the primary outcome of 90-day new ischemic stroke (HR 2.04, 95% CI 1.18–3.53; *p* = 0.011) compared with those without potential causes of OAC failure (Table 2). Rates of secondary and safety outcomes did not differ between the two groups (Table 2). The between-group difference in the cumulative incidence of the primary outcome was also found by the log-rank test (Fig. 1).

**Table 2:** Ninety-day outcomes in the overall cohort and in patients with and without causal factors for breakthrough ischemic stroke despite oral anticoagulation. All data are reported as numbers and proportions

	Overall (*n* = 1649)	No potential cause (*n* = 925, 56.1%)	≥ 1 potential cause (*n* = 724, 43.9%)	Adjusted HR* (95% CI)	*P* value
Primary					
90-day new ischemic stroke	57 (3.5)	22 (2.4)	35 (4.8)	2.04 (1.18 − 3.53)	**0.011**
Secondary					
90-day mRS score distribution				1.15 (0.97 − 1.37)**	0.106
0	194 (11.8)	117 (12.6)	77 (10.6)		
1	284 (17.2)	167 (18.1)	117 (16.2)		
2	239 (14.5)	125 (13.5)	114 (15.7)		
3	265 (16.1)	153 (16.5)	112 (15.5)		
4	217 (13.2)	116 (12.5)	101 (14.0)		
5	16 (7.0)	77 (8.3)	39 (5.4)		
6	334 (20.3)	170 (18.4)	164 (22.7)		
90-day myocardial infarction	21 (1.3)	10 (1.1)	11 (1.5)	1.70 (0.69 − 4.14)	0.246
90-day all-cause death	334 (20.3)	170 (18.4)	164 (22.7)	1.24 (0.99 − 1.55)	0.057
90-day vascular death	215 (13.0)	116 (12.5)	99 (13.7)	1.06 (0.80 − 1.40)	0.697
Safety					
90-day moderate-to-severe bleeding	55 (3.3)	26 (2.8)	29 (4.0)	1.38 (0.80 − 2.38)	0.245
90-day intracranial hemorrhage	28 (1.7)	13 (1.4)	15 (2.1)	1.48 (0.69 − 3.18)	0.310

^*^Adjusted for age, sex, arterial hypertension, smoking, diabetes mellitus, dyslipidemia, diabetes mellitus, prior myocardial infarction, prior ischemic stroke or TIA, and prior intracerebral hemorrhage

^**^Odds ratio for ordinal analysis

### Comparison between patients taking and those not taking drugs interacting with OACs

A comparison of baseline characteristics between patients taking and not taking drugs interacting with OACs for the unweighted population is reported in Supplementary Table 3. The IPW resulted in the comparison between 818 patients taking and 831 not taking interacting drugs at the time of the index event (Table 3). The two groups were well balanced after IPW, as shown by the SMD values (Supplementary Table 3) and by the balance plot (Supplementary Fig. 2).

**Table 3:** Ninety-day outcomes in patients taking and not taking drugs interacting with oral anticoagulation

	No interacting drugs (*n* = 831)	Interacting drugs (*n* = 818)	HR (95% CI)	*P* value
Primary				
90-day new ischemic stroke	33 (4.0)	35 (4.3)	1.08 (0.67 − 1.73)	0.761
Secondary				
90-day mRS score distribution			1.10 (0.93 − 1.30)	0.282
0	91 (11.0)	103 (12.6)		
1	161 (19.4)	122 (14.9)		
2	130 (15.6)	136 (16.6)		
3	137 (16.5)	134 (16.4)		
4	106 (12.8)	103 (12.6)		
5	63 (7.6)	48 (5.9)		
6	143 (17.2)	172 (21.0)		
90-day myocardial infarction	6 (0.7)	19 (2.3)	3.26 (1.30 − 8.17)	**0.012**
90-day all-cause death	143 (17.2)	172 (21.0)	1.22 (0.98 − 1.53)	0.076
90-day vascular death	98 (11.8)	80 (9.8)	0.83 (0.62 − 1.11)	0.209
Safety				
90-day moderate-to-severe bleeding	21 (2.5)	27 (3.3)	1.33 (0.75 − 2.34)	0.333
90-day intracranial hemorrhage	6 (0.7)	15 (1.8)	2.56 (0.99 − 6.60)	0.052

^*^Odds ratio for ordinal analysis

Data are from the inverse probability-weighted population. All data are reported as numbers and proportions. Hazard ratios are unadjusted to avoid overfitting as they are based upon the inversion probability-weighted population

The comparison of 90-day outcomes between patients taking and those not taking drugs interacting with OACs in the weighted population showed no difference in the primary outcome (Table 3). Among secondary outcomes, we found a significantly increased risk of 90-day myocardial infarction in patients taking compared with those not taking drugs interacting with OACs at the time of the index event (HR 3.26, 95% CI 1.30–8.17, *p* = 0.012; Table 3).

## Discussion

The ASPERA-R study reported real-world data on the characteristics and management of breakthrough ischemic stroke occurring in patients with ongoing OAC therapy in contemporary clinical practice, where patients with non-valvular AF are mostly treated with DOACs rather than VKAs [19]. The main goal of the present report was to describe the occurrence and outcome of known potential cause of breakthrough ischemic stroke in a cohort of patients reporting full adherence and correct dosing to OAC intake and to provide a thorough focus on interacting drugs that was not yet covered elsewhere yet. At variance with previous reports on the ASPERA-R cohort, we focused on descriptive data from the whole population to provide the prevalence of causes of OAC failure and their overlap.

We observed that nearly half of our population had potential causes that may have contributed to breakthrough ischemic stroke. Besides, those potential causes also had an impact on clinical outcomes, as their presence conferred a twofold risk for new ischemic stroke compared with those without, in line with previous evidence [23]. However, when focusing on patients taking drugs potentially interacting with OACs, this potential cause of breakthrough ischemic stroke was not associated with an increased risk of ischemic stroke, being associated with an increased risk of myocardial infarction at 90 days after the index event.

The finding of the present study may have some important implications. Upstream identification and management of factors that may contribute to breakthrough ischemic stroke may prevent the occurrence of this condition. Moreover, after the breakthrough event, proper management of those factors may improve secondary stroke prevention. Trials on breakthrough ischemic stroke are already ongoing [24–26] and others may be planned in the future. Having information on factors potentially contributing to breakthrough ischemic stroke may be helpful to properly select the population and the interventions to be included in those trials, to properly consider confounders and to plan subgroup analyses.

We found that nearly half of the patients with breakthrough ischemic stroke had a potential cause for that event. This may represent an underestimation of currently identifiable causes of breakthrough ischemic stroke, since some mechanisms—such as malabsorption syndromes, food interactions, rare hypercoagulable states, or chronic inflammatory conditions—were not captured in the ASPERA-R cohort. Nevertheless, previous studies focusing on the potential causes of OAC failure [23, 27] also ignored those mechanisms, due to lack of specific data.

Concurrent intake of medications with known interaction with OACs was the most frequent potential cause of OAC failure (28.4%), followed by competing stroke etiologies different from AF-related cardioembolism (24.3%) and by the presence of active cancer (4.4%). Notably, there was a partial overlap among potential causes of breakthrough ischemic stroke, with a small proportion of patients presenting more than one potential cause. The impact of specific factors such as cancer and competing stroke etiologies has been previously reported in the ASPERA-R cohort. [20, 21] However, those analyses were restricted to individual mechanisms of breakthrough ischemic stroke. In contrast, the present study provides a comprehensive and integrated assessment of all potential causes within the same population, allowing, for the first time, an estimation of their relative prevalence, co-occurrence, and combined impact on outcomes. This broader approach is essential, as breakthrough ischemic stroke cannot be fully understood through isolated analyses of single determinants.

Referring to interacting drugs potentially impairing the efficacy and safety of OACs, we expanded the knowledge compared to the already available information, as we considered all potential drug classes interacting with the cytochrome P function, including antifungal agents, chemotherapeutics, anti-seizure medications, and proton pump inhibitors. This might explain why we found a slightly higher (28.4%) prevalence as compared to what was already reported [23]. Notably, all interacting drugs found in our study were cytochrome P inducers and therefore expected to decrease the anticoagulant activity of both DOACs and VKAs. Previous observational studies reported an increased risk of mortality [28], ischemic stroke [29], bleeding [30], or both [31] in patients with AF taking drugs interacting with OACs. The interaction of proton pump inhibitors with OACs was studied mostly in patients treated with dabigatran [16, 32, 33] and warfarin [34, 35]. Proton pump inhibitors represented the vast majority of drugs interacting with OACs that were identified in our study, while the remaining interacting drugs were taken by few patients, highlighting the awareness of clinicians about some classes of drugs—especially antiseizure medications—that could interact with OACs. Notably, although we included any drug with potential interaction with OACs, literature is not consistent about the interaction with proton pump inhibitors [36] and even with antiseizure medication [37]. In clinical practice, it can be useful to periodically review the co-prescriptions of patients treated with OACs and particularly those with breakthrough ischemic stroke, to minimize the risk of OAC failure due to drug-drug interactions. For patients’ safety, it might be advisable to only keep drugs whose prescription is strictly necessary. Although the potential for interaction of drugs such as proton pump inhibitors is probably limited and the evidence is conflicting, it cannot be taken as non-existent. Variability of anticoagulant effect, potentially translating into an increased bleeding risk through cytochrome P pharmacokinetic interactions, was described especially for VKAs [38], while among DOACs reduced absorption associated with increased gastric pH has mainly been reported for dabigatran [39]. Other studies comparing patients treated with those not treated with proton pump inhibitors found no significant differences in DOAC plasma levels between the two patient groups [40] or reported an overall increase in mortality risk without significant differences in thrombotic or bleeding events [36]. Additionally, patients treated with OACs are often on polypharmacy, which can further complicate the issues of drug–drug interactions. On the other hand, some recommendations suggest the use of proton pump inhibitors to decrease the risk of gastrointestinal bleeding in patients taking OACs and particularly in those with high risk for bleeding events [41]. It is relevant to note that in our study, patients treated with drugs interacting with OACs did not have an increased risk for recurrent ischemic stroke or bleeding events at the 90-day follow-up, while only reporting a slightly increased risk for myocardial infarction (Table 3). This finding might suggest that the use of most drugs with potential interactions with OACs are safe; however, the short follow-up and limited number of outcome events in our population might have hindered some unfavorable outcomes for patients treated with drugs interacting with OACs.

The outcome rates of our study should be evaluated in the context of therapeutic adherence to OAC prescription, distinguishing ASPERA-R from previous observational studies. Our 3.5% rate of 90-day new ischemic stroke was lower than the 6.9% of 90-day recurrent ischemic events—both cerebral and cardiac – found by a previous study [42] and even lower than the 5% rate of new embolic recurrence or severe bleeding at 90 days found by a large retrospective study [43]. The relatively low prevalence of early ischemic recurrences in our population can be explained by a high competing fatality of 20.3%, which was much higher than a previous pooled analysis of randomized controlled trials (12.4%) [44]. However, our fatality rate was in line with a large retrospective real-world study of patients included from Germany, Switzerland, and the USA—including many elderly patients like our study—which showed a rate of 90-day recurrent ischemic stroke of 4.6% and an all-cause death rate of 22.8% [23]. The cumulative incidence of recurrent ischemic stroke was comparable to that of moderate-to-severe bleeding (Table 2), suggesting that both ischemic and hemorrhagic events might have contributed to the disability and high fatality rate found in our study.

Taking all together, our findings have several clinical implications. The high proportion of patients taking concomitant drugs interacting with OACs should encourage clinicians to pay attention to polypharmacy. Active cancer might also impair the efficacy of OACs, while the high prevalence of non-cardioembolic etiologies of breakthrough ischemic stroke should prompt clinicians to perform an extensive diagnostic workup in those patients and adopting secondary prevention strategies that go beyond anticoagulation. Our results highlight the need for personalized secondary prevention approaches to patients with breakthrough stroke including statins, antiplatelet treatments, and/or other drugs, as breakthrough stroke might depend in many cases on alternative stroke mechanisms rather than on insufficient anticoagulation.

The strengths of this study include its large sample size and the application of rigorous procedures to ensure data accuracy and quality, reinforced by systematic quality checks of the ASPERA-R electronic database. Nevertheless, several limitations should be acknowledged. The retrospective nature of the cohort carries inherent risks of selection and information bias, as data collection relied on hospital records and follow-up information was partly reconstructed. Compliance to DOACs was retrieved from clinical history only, in the absence of objective methods used by all participating centers. Adjudication of outcome events was performed by the local investigators without a centralized check by the study coordinating committee. As clinical procedures were not mandatory, some patients might have lacked diagnostic tests such as echocardiography in the diagnostic workup of breakthrough ischemic stroke or its recurrences, with a potential effect on the adjudication of stroke causes. Additionally, some items of remote medical history might have been underestimated because of recall bias. We did not collect information on the adherence and compliance to secondary preventive strategies including both OACs and other treatments, which might have played a role in the genesis of non-cardioembolic breakthrough strokes. Moreover, the 1.5 INR cutoff for inclusion of patients treated with VKAs might have led to the inclusion of some patients with incomplete adherence to those drugs. We also did not collect information about the possible discontinuation of drugs interacting with OACs after the index event, which might have had an impact on patients’ outcome. Besides, we could not quantify the action of causal factors on the plasma concentration of OACs, as we did not include mandatory laboratory collections to test OAC effectiveness in our purely observational study. Furthermore, most patients were recruited in Italy (60.0%), which might limit generalizability as our findings apply to a population predominantly composed of non-Hispanic white individuals.

## Conclusions

Our real-world study shows that a substantial proportion of patients who experience breakthrough ischemic stroke despite ongoing OAC for AF have identifiable and partly modifiable causes of breakthrough ischemic stroke, most commonly drug–drug interactions, non-cardioembolic stroke mechanisms, and active cancer. These findings highlight that systematic identification and proactive management of these factors may represent an opportunity to prevent breakthrough strokes. At the same time, careful evaluation of these mechanisms at the time of breakthrough stroke is essential to optimize secondary prevention and reduce the risk of recurrence and adverse outcomes. Breakthrough stroke emerges as a heterogeneous, often multifactorial condition, that cannot be adequately addressed with a uniform therapeutic approach. Future interventional trials should explicitly account for this complexity in patients’ selection and incorporating mechanism-driven subgroup analyses. Tailored prevention strategies will be required, as optimal management is likely to differ across distinct patient profiles.

## Electronic supplementary material

Below is the link to the electronic supplementary material.

Supplementary file1 (DOCX 825 KB)

## Data Availability

The ASPERA-R dataset is available from the corresponding author upon reasonable request.

## Appendix 1. Distribution of patients by country in the ASPERA-R

**Table:**

Country	Enrolling center(s)	No. of patients/total no. included in the ASPERA-R (%)	No. of patients/total no. included in the study analysis (%)
Italy	• Stroke Unit and Neurology Unit, S.S. Filippo e Nicola Hospital, Avezzano • Stroke Unit, Maggiore Hospital, Bologna • Neurology Unit, IRCCS Policlinico S.Orsola-Malpighi, Bologna • Stroke Unit, IRCCS Policlinico Universitario Agostino Gemelli, Rome • Stroke Unit—Neurology Unit, S.Maria delle Croci Hospital, Ravenna • Stroke Unit, ASST Grande Ospedale Metropolitano Niguarda, Milano • Stroke Unit, Luigi Sacco Hospital, Milano • Stroke Unit, Policlinico San Matteo, Pavia • Stroke Unit – Neurology Unit, Vito Fazzi Hospital, Lecce • Stroke Unit, Fabrizio Spaziani Hospital, Frosinone • Stroke Unit, S.Maria della Misericordia Hospital, Perugia • Stroke Unit, Azienda Ospedaliera Ospedali Riuniti Villa Sofia – Cervello, Palermo • Stroke Unit, Arcispedale Santa Maria Nuova, Reggio Emilia • Stroke Unit, AORN Cardarelli Hospital, Napoli • Stroke Unit, Azienda Ospedaliero Universitaria Careggi, Firenze • Stroke Unit, S.Maria della Misericordia Hospital, Udine • Stroke Unit, Ospedale Riuniti di Ancona, Ancona • Stroke Unit, Maggiore Hospital, Crema • Stroke Unit—Neurology Unit, “Di Venere” Hospital, Bari	1010/1772 (57.0)	989/1649 (60.0)
France	• Stroke Unit, Université Côte d’Azur, Nice	79/1772 (4.5)	78/1649 (4.7)
Germany	• Stroke Unit—Neurology Unit, Martin Luther University, Halle-Wittenberg • Stroke Unit, Saarland University Hospital, Homburg	67/1772 (3.8)	43/1649 (2.6)
Spain	• Stroke Unit, Hospital Universitario La Paz, Madrid	81/1772 (4.6)	80/1649 (4.9)
Portugal	• Stroke Unit, São José Hospital, Lisbon • Stroke Unit, Hospital de Santa Maria, Lisbon	124/1772 (7.0)	101/1649 (6.1)
United Kingdom	• Stroke Unit, Charing Cross Hospital, London • Stroke Unit, St George University Hospital, London • Stroke Unit, North Bristol NHS Trust, Bristol	180/1772 (10.2)	178/1649 (10.8)
Croatia	• Stroke Unit, Sveti Duh University Hospital, Zagreb	35/1772 (19.7)	32/1649 (19.4)
Egypt and Saudi Arabia	• Stroke Unit – Neurology Unit, Aim Shams University Hospital, Cairo • Stroke Unit, Armed Forces Medical Complex Kobry El Kobba, Cairo • Stroke Unit, Cairo University Hospital, Cairo • Stroke Unit, Assiut University Hospital, Asyut • Stroke Unit—Neurology Unit, King Fahd Hospital, Riyadh	196/1772 (11.1)	148/1949 (9.0)

## Appendix 2. List of baseline variables collected in the ASPERA-R study

**Table:**

Variable	Mandatory	Notes
Demographics		
Date of index ischemic stroke on oral anticoagulation	Yes	–
Hospitalization (Yes/No)	Yes	–
Hospitalization setting - Stroke unit - Intensive care unit - Other hospital unit - Emergency department only	Yes	–
Date of admission	Yes	–
Sex - Male - Female - Other	Yes	–
Ethnicity - Non-Hispanic white - Hispanic white - Black - Asian - Other	Yes	–
Date of birth	Yes	–
Weight—kg	No	–
Height—cm	No	–
Risk factors		
Current cigarette smoking (Yes/No)	Yes	Consumption of ≥ 1 cigarette per day over the last year
Arterial hypertension (Yes/No)	Yes	Blood pressure of ≥ 140/90 mmHg at least twice before stroke or already under treatment with antihypertensive drugs
Dyslipidemia (Yes/No)	Yes	History of total blood cholesterol levels > 220 mg/dL and/or total triglycerides levels > 130 mg/dL and/or current used lipid-lowering drugs
Diabetes (Yes/No)	Yes	History of fasting glucose > 126 mg/dL or the current use of hypoglycemic medications
Ischemic heart disease (Yes/No)	Yes	History of myocardial infarction, angina or prior evidence of coronary disease on coronary angiography
Chronic congestive heart failure (Yes/No)	Yes	History of stage C (structural heart disease and current or past history of heart-failure symptoms) or stage D (refractory symptoms that interfere with daily life or recurrent hospitalization despite targeted guideline-directed medical therapy) chronic heart failure
Chronic kidney disease (Yes/No)	Yes	History of estimated creatinine clearance of less than 60 for 3 months or more (including dialysis)
Chronic liver failure (Yes/No)	Yes	History of cirrhosis or end-stage liver disease
Symptomatic peripheral artery disease (Yes/No)	Yes	History of intermittent claudication of presumed atherosclerotic origin
Prior ischemic stroke or TIA (Yes/No)	Yes	–
Prior intracranial hemorrhage (Yes/No)	Yes	–
History of malignancy (Yes/No)	Yes	–
Type of malignancy - Active - In remission	Yes	We defined active malignancy as (1) a diagnosis of cancer that occurred within 6 months of the index event or during hospitalization, (2) cancer treatment with radiotherapy, chemotherapy, or surgery or a combination of them within 6 months of the index event, (3) a previous history of malignancy and a diagnosis of recurrence or metastasis within 6 months of the index event. In remission was defined as a previous history of malignancy in the absence of active cancer criteria
Site of malignancy - Gastrointestinal - Lung - Genitourinary - Breast - Hematological - Skin - Other	Yes	–
Metastatic malignancy? (Yes/No)	Yes	–
Metastasis location - Lymph nodes - Liver - Lung - Bones - Brain/spine/meningeal - Other	Yes	–
Atrial fibrillation type - Paroxysmal - Persistent - Long-standing persistent - Permanent	Yes	According to the ACC/AHA/HRS guidelines classification
History of valvular heart disease (multiple choice) - Severe mitral stenosis - Severe aortic stenosis - Severe mitral insufficiency - Severe aortic insufficiency - Mechanical heart valve - Biological heart valve	Yes	–
Pacemaker (Yes/No)	Yes	–
Left atrial volume index at transthoracic echocardiography—mL/m^2^	No	–
Left ventricle end-diastolic volume at transthoracic echocardiography—mL	No	–
Left ventricle end-systolic volume at transthoracic echocardiography—mL	No	–
Left ventricle ejection fraction at transthoracic echocardiography—mL	No	–
Drugs history		
Type of oral anticoagulant ongoing at the time of the index ischemic stroke - Vitamin K antagonist - Direct oral anticoagulant	Yes	–
Type of direct oral anticoagulant - Apixaban - Rivaroxaban - Edoxaban - Dabigatran	Yes	–
Type of vitamin K antagonist - Warfarin - Acenocoumarol - Other	Yes	–
Time from last direct oral anticoagulant intake to admission - < 12 h - 12–24 h - 24–48 h	Yes	–
Direct oral anticoagulant plasma levels or anti-factor Xa activity available on admission	Yes	–
Direct oral anticoagulant plasma levels (ng/mL) or anti-factor Xa activity on admission	Yes	–
Direct oral anticoagulant plasma levels or anti-factor Xa activity on admission - Below range - Within range - Above range	Yes	In respect to the range locally determined for therapeutic anticoagulation
Direct oral anticoagulant at reduced dose at the time of the index ischemic stroke (Yes/No)	Yes	Apixaban 2.5 mg BID, dabigatran 75 mg BID, edoxaban 30 mg daily, rivaroxaban 15 mg daily
Direct oral anticoagulant reduced dose appropriate (Yes/No)	Yes	Reduced dose of apixaban was considered appropriate if 2 of 3 factors were present: 1) age ≥ 80 years; 2) serum creatinine ≥ 1.5 mg/dL; 3) weight ≤ 60 kg OR if apixaban is co-administered with combined P-gp and strong CYP3A4 inhibitors (e.g., ketoconazole, itraconazole, ritonavir). Reduced dose of dabigatran was considered appropriate if creatinine clearance 15–30 mL/min OR, creatinine clearance 30–50 mL/min with concomitant dronedarone or ketoconazole. Reduced dose of edoxaban was considered appropriate if creatinine clearance 15–50 mL/min. Reduced dose of rivaroxaban was considered appropriate if creatinine clearance ≤ 50 mL/min
Antihypertensive drugs on admission (Yes/No)	Yes	–
Lipid-lowering drugs on admission (Yes/No)	Yes	–
Antidiabetic drugs on admission (Yes/No)	Yes	–
Rhythm control drugs on admission (Yes/No)	Yes	–
Type of rhythm control drugs on admission - Class I—sodium channel blockers - Class II—beta-blockers - Class III—potassium channel blockers - Class IV—calcium channel blockers - Class V—miscellaneous agents (i.e., digoxin, ivabradine, adenosine)	Yes	According to the Vaughan Williams classification of antiarrhythmic drugs
Drugs potentially interfering with oral anticoagulation at the time of the index stroke (multiple choice) - Itraconazole - Ketoconazole - Clarithromycin - Lopinavir - Indinavir - Ritonavir - Telaprevir - Voriconazole - Any H2 inhibitor (i.e., cimetidine) - Any proton pump inhibitor (i.e., omeprazole, pantoprazole) - Doxorubicin - Vinblastine - Carbamazepine/oxcarbazepine - Phenytoin - Phenobarbital - Rifampin - Levetiracetam - Valproic acid - Dexamethasone - Tocilizumab		–
Antiplatelet therapy on admission (Yes/No)	Yes	–
Type of antiplatelet therapy on admission (multiple choice) - Aspirin - Clopidogrel - Ticagrelor - Ticlopidine - Other	Yes	–
Antihypertensive drugs on admission (Yes/No)	Yes	–
Clinical characteristics		
Modified Rankin Scale (mRS) score before the index ischemic stroke	Yes	–
Type of ischemic stroke onset - Known onset - Wake-up stroke - Unwitnessed stroke	Yes	–
National Institute of Health Stroke Scale (NIHSS) score on admission (prior to any acute reperfusion therapy)	Yes	–
Acute ischemic stroke symptoms (multiple choice) - Motor weakness - Aphasia - Dysarthria - Sensory defect - Visual field defect - Diplopia - Vertigo - Loss of balance - Hemineglect - Other	No	–
Clinical classification of the acute ischemic stroke - Total anterior circulation stroke (TACS) - Partial anterior circulation stroke (PACS) - Posterior circulation syndrome (PoCS) - Lacunar stroke (LACS)	Yes	According to the Oxfordshire Stroke Project Classification
Admission systolic blood pressure—mmHg	No	–
Admission diastolic blood pressure—mmHg	No	–
Admission Glasgow Coma Scale (GCS) score	No	–
Presence of competing stroke etiology other than cardioembolism (Yes/No)	Yes	–
Type of competing stroke etiology other than cardioembolism - Lacunar - Large artery atherosclerosis - Other determined etiology	Yes	According to the Trial of Org 10172 in the Acute Stroke Treatment (TOAST) classification system
Type of other determined etiology (specify)	Yes	–
Competing cardioembolic mechanisms other than atrial fibrillation (Yes/No)	Yes	–
Type of competing cardioembolic mechanisms other than atrial fibrillation (multiple choice) - Reduced left ventricle ejection fraction (30–40%) - Severely reduced left ventricle ejection fraction (< 30%) - Left ventricle thrombus - Endocarditis - Mechanical heart valve - Biological heart valve - Atrial myxoma - Fibroelastoma - Other cardiac tumors - Patent foramen ovale (PFO) - Interventricular defect (IVD) - Left ventricular aneurysm - Left atrial aneurysm - Other cardiac congenital alterations	Yes	–
Intravenous thrombolysis (Yes/No)	Yes	–
Endovascular thrombectomy (Yes/No)	Yes	–
Onset-to-needle time—min	Yes	
Onset-to-groin time—min	Yes	
Hemorrhagic infarction - Yes—asymptomatic - Yes—symptomatic - No	Yes	Symptomatic hemorrhagic infarction was defined according to SITS-MOST as a neurologic deterioration of ≥ 4 NIHSS points or leading to death within 24 h
Hemorrhagic infarction type (multiple choice) - HI1: scattered small petechiae, no mass effect - HI2: confluent petechiae, no mass effect - PH1: hematoma within infarcted tissue, occupying < 30%, no substantive mass effect - PH2: hematoma occupying 30% or more of the infarcted tissue, with obvious mass effect PH remote from infarcted brain tissue - rPH: remote parenchymal hematoma - Intraventricular hemorrhage - Subarachnoid hemorrhage - Subdural hemorrhage	Yes	According to the Heidelberg classification system
National Institute of Health Stroke Scale (NIHSS) score at 24 h	No	–
Neuroimaging information		
Type of brain neuroimaging performed on admission - Non-contrast computed tomography - Magnetic resonance imaging - Both	Yes	–
Type of brain neuroimaging performed at 24 h follow-up - Non-contrast computed tomography - Magnetic resonance imaging - Both - None	Yes	–
Type of brain vessel imaging performed (multiple choice) - Computed tomography angiography—extracranial vessels - Computed tomography angiography—intracranial vessels - Magnetic resonance angiography—extracranial vessels - Magnetic resonance angiography—intracranial vessels - Color Doppler ultrasonography—extracranial vessels - Color Doppler ultrasonography—intracranial vessels - X-ray angiography - None	Yes	–
Large vessel occlusion (Yes/No/Unknown)	Yes	–
Large vessel occlusion site (multiple choice) - Middle cerebral artery—M1 - Middle cerebral artery— M2 - Middle cerebral artery—more distal than M2 - Tandem occlusion - Anterior cerebral artery - Posterior cerebral artery - Basilar artery - Internal carotid artery - Vertebral artery	Yes	–
Baseline modified Thrombolysis in Cerebral Infarction score - Grade 0: no perfusion - Grade 1: antegrade reperfusion past the initial occlusion, but limited distal branch filling with little or slow distal reperfusion - Grade 2a: antegrade reperfusion of less than half of the occluded target artery previously ischemic territory (i.e., in one major division of the middle cerebral artery and its territory) - Grade 2b: antegrade reperfusion of more than half of the previously occluded target artery ischemic territory (i.e., in two major divisions of the MCA and their territories) - Grade 2c: near complete perfusion except for slow flow or distal emboli in a few distal cortical vessels - Grade 3: complete antegrade reperfusion of the previously occluded target artery ischemic territory, with absence of visualized occlusion in all distal branches	Yes	–
Post-endovascular thrombectomy modified Thrombolysis in Cerebral Infarction score - Grade 0: no perfusion - Grade 1: antegrade reperfusion past the initial occlusion, but limited distal branch filling with little or slow distal reperfusion - Grade 2a: antegrade reperfusion of less than half of the occluded target artery previously ischemic territory (i.e., in one major division of the middle cerebral artery and its territory) - Grade 2b: antegrade reperfusion of more than half of the previously occluded target artery ischemic territory (i.e., in two major divisions of the MCA and their territories) - Grade 2c: near complete perfusion except for slow flow or distal emboli in a few distal cortical vessels - Grade 3: complete antegrade reperfusion of the previously occluded target artery ischemic territory, with absence of visualized occlusion in all distal branches	Yes	–
Presence of extracranial internal artery stenosis ≥ 50% ipsilateral to the acute ischemic stroke lesion (Yes/No)	Yes	–
Degree of extracranial internal artery stenosis ≥ 50% ipsilateral to the acute ischemic stroke lesion - 50–69% - 70–79% - 80–99% - Occlusion (100%)	Yes	–
Presence of extracranial vertebral artery stenosis ≥ 50% ipsilateral to the acute ischemic stroke lesion (Yes/No)	Yes	–
Degree of extracranial vertebral artery stenosis ≥ 50% ipsilateral to the acute ischemic stroke lesion - 50–69% - 70–79% - 80–99% - Occlusion (100%)	Yes	–
Presence of intracranial artery stenosis (Yes/No)	Yes	–
Site of intracranial artery stenosis (multiple choice) - Middle cerebral artery - Anterior cerebral artery - Posterior cerebral artery - Basilar artery - Internal carotid artery - Vertebral artery	Yes	–
Presence of symptomatic intracranial artery stenosis (Yes/No)	Yes	–
Laboratory tests		
International normalized ratio (INR)	Yes	–
aPTT—seconds	No	–
PT—seconds	No	–
Red blood cells—millions/mm^3^	No	–
White blood cells—millions/mm^3^	No	–
Lymphocytes—millions/mm^3^	No	–
Neutrophiles—millions/mm^3^	No	–
Platelets—thousands/mm^3^	No	–
Hemoglobin—mg/dL	No	–
Admission blood glucose levels—mg/dL	No	–
Fasting blood glucose levels—mg/dL	No	–
C-reactive protein—mg/dL	No	–
Creatinine—mg/dL	No	–
Creatinine clearance—mL/min	No	–
Glycate hemoglobin—mmol/L	No	–
Total cholesterol—mg/dL	No	–
High density lipoprotein (HDL)—mg/dL	No	–
Low density lipoprotein (LDL)—mg/dL	No	–
Triglycerides—mg/dL	No	–
ALT/GPT—U/L	No	–
AST/GOT—U/L	No	–
Gamma-GT—U/L	No	–
Total bilirubin—mg/dL	No	–
