# Supplementary material for: Decoding the causes of ischemic stroke despite ongoing oral anticoagulation and their clinical impact: the ASPERA-R study
Source: J Neurol. 2026 Aug 17;273(9):526. doi: 10.1007/s00415-026-14039-x (PMC13478031; doi:10.1007/s00415-026-14039-x)
Supplement: Supplementary file 1 [file 415_2026_14039_MOESM1_ESM.docx]

**Supplemental material**

**Supplementary Figure 1. Flow-chart of patients included in ASPERA-R**

**2546** patients with neuroimaging-confirmed ischemic stroke receiving oral anticoagulation for atrial fibrillation screened across 35 centers between February 2020 and February 2025

**899** patients excluded:

• 341 poor adherence to anticoagulant therapy

• 273 inadequate anticoagulant dosing

• 162 baseline extracranial or intracranial vascular imaging not available

• 92 incomplete data for 21 mandatory baseline variable

• 31 missing 90-day follow-up or unrecorded study outcomes

**1649** included in the present analysis

**Supplementary Table 1.** Details on drugs interacting with oral anticoagulants.

| **Drug** | **DOACs (n=1275); n (%)** | **VKA (n=374); n (%)** |
| --- | --- | --- |
| Ketoconazole | 1 (0.1) | - |
| H2 inhibitors | 3 (0.2) | - |
| Proton pump inhibitors | 335 (26.3) | 115 (30.7) |
| Carbamazepine | 4 (0.3) | - |
| Phenytoin | 2 (0.2) | - |
| Phenobarbital | 2 (0.2) | 1 (0.3) |
| Levetiracetam | 14 (1.1) | 3 (0.8) |
| Oxcarbazepine | 2 (0.2) | - |
| Dexamethasone | 2 (0.2) | 1 (0.3) |

**Supplementary Table 2**. Comparison of the baseline characteristics among patients with one potential cause for breakthrough ischemic stroke, those with more than one potential cause, and no identified cause.

|  | **No potential cause**  **(n=925)** | **1 potential cause**  **(n=544)** | **p value (1 vs no potential cause)** | **>1 potential cause**  **(n=180)** | **p-value (>1 vs no potential cause)** | **p value (>1 vs 1 potential cause)** |
| --- | --- | --- | --- | --- | --- | --- |
| Age (median-[IQR]) | 80.5 (73.3-85.6) | 80.0 (72.4-85.5) | >0.999 | 80.5 (75.2-85.2) | >0.999 | 0.810 |
| Female, n (%) | 489 (52.9) | 278 (47.8)) | 0.849 | 93 (51.7) | 0.302 | 0.415 |
| Ethnicity, n (%) |  |  | 0.066 |  | **<0.001** | **0.002** |
| Caucasian | 718 (77.6) | 442 (81.2) |  | 143 (79.4) |  |  |
| Hispanic white | 52 (5.6) | 37 (6.8) |  | 24 (13.3) |  |  |
| Black | 17 (1.8) | 3 (0.6) |  | 4 (2.2) |  |  |
| Asian | 5 (0.5) | 3 (0.6) |  | 1 (0.6) |  |  |
| Other | 133 (14.4) | 59 (10.8) |  | 8 (4.4) |  |  |
| Hospitalized |  |  | 0.911 |  | 0.742 | 0.826 |
| Stroke unit | 842 (94.5) | 497 (94.0) |  | 165 (93.8) |  |  |
| Intensive care unit | 23 (2.6) | 15 (2.8) |  | 4 (2.3) |  |  |
| Other hospital unit | 26 (2.9) | 17 (3.2) |  | 7 (4.0) |  |  |
| Risk factors (n, %): |  |  |  |  |  |  |
| Smoking | 89 (9.6) | 53 (9.7) | >0.999 | 12 (6.7) | 0.264 | 0.271 |
| Hypertension | 706 (76.3) | 477 (87.7) | **<0.001** | 155 (86.1) | **0.005** | 0.674 |
| Dyslipidemia | 424 (45.8) | 311 (57.2) | **<0.001** | 110 (61.1) | **<0.001** | 0.400 |
| Diabetes | 225 (24.3) | 162 (29.8) | **0.026** | 56 (31.1) | 0.069 | 0.807 |
| Prior Myocardial Infarction | 154 (16.6) | 156 (28.7) | **<0.001** | 56 (31.1) | **<0.001** | 0.598 |
| Prior Stroke/TIA | 202 (21.8) | 155 (28.5) | **0.005** | 53 (29.4) | **0.034** | 0.881 |
| Prior ICH | 13 (1.4) | 11 (2.0) | 0.492 | 3 (1.7) | >0.999 | >0.999 |
| Chronic heart failure | 124 (13.4) | 114 (21.0) | **<0.001** | 39 (21.7) | **0.006** | 0.923 |
| Chronic liver failure | 2 (0.2) | 3 (0.6) | 0.548 | 2 (1.1) | 0.250 | 0.790 |
| Chronic kidney failure | 120 (13.0) | 105 (19.3) | **0.001** | 43 (23.9) | **<0.001** | 0.224 |
| Peripheral artery disease | 27 (2.9) | 33 (6.1) | **0.005** | 20 (11.1) | **<0.001** | **0.037** |
| Valvular heart disease |  |  | 0.431 |  | 0.239 | 0.869 |
| Mitral stenosis | 19 (2.1) | 12 (2.2) |  | 2 (1.1) |  |  |
| Aortic stenosis | 11 (1.2) | 14 (2.6) |  | 6 (3.3) |  |  |
| Mitral insufficiency | 24 (2.6) | 16 (2.9) |  | 6 (3.3) |  |  |
| Aortic insufficiency | 5 (0.5) | 2 (0.4) |  | - |  |  |
| Multiple | 7 (0.8) | 6 (1.1) |  | 2 (1.1) |  |  |
| Mechanical valve replacement | 46 (5.0) | 38 (7.0) | 0.137 | 10 (5.6) | 0.888 | 0.620 |
| Biological valve replacement | 38 (4.1) | 30 (5.5) | 0.267 | 13 (7.2) | 0.104 | 0.510 |
| Pacemaker | 103 (11.1) | 76 (14.0) | 0.128 | 32 (17.8) | **0.018** | 0.261 |
| Atrial fibrillation type |  |  | **<0.001** |  | **<0.001** | 0.156 |
| Paroxysmal | 158 (17.1) | 122 (22.4) |  | 55 (30.6) |  |  |
| Persistent | 129 (13.9) | 59 (10.8) |  | 12 (6.7) |  |  |
| Long-standing persistent | 43 (4.6) | 26 (4.8) |  | 7 (3.9) |  |  |
| Permanent | 454 (49.1) | 300 (55.1) |  | 93 (51.7) |  |  |
| Not classified | 141 (15.2) | 37 (6.8) |  | 13 (7.2) |  |  |
| Anticoagulation type |  |  | 0.542 |  | 0.752 | 0.473 |
| VKA | 213 (23.1) | 117 (21.5) |  | 44 (24.4) |  |  |
| DOAC | 712 (77.0) | 427 (78.5) |  | 136 (75.6) |  |  |
| Minor stroke | 247 (26.7) | 181 (33.3) | **0.009** | 64 (35.6) | **0.020** | 0.638 |
| Minor to moderate stroke | 430 (46.5) | 277 (50.9) | 0.112 | 94 (52.2) | 0.184 | 0.828 |
| NIHSS baseline (median) | 12 (5-18) | 10 (4-18) |  | 10 (3-17) |  |  |
| Large vessel occlusion | 533 (57.6) | 285 (52.4) | **0.013** | 94 () | 0.097 | 0.953 |
| IVT | 81 (8.8) | 49 (9.0) | 0.946 | 9 (5.0) | 0.124 | 0.119 |
| EVT | 436 (47.1) | 222 (40.8) | **0.021** | **74 (41.1)** | 0.161 | >0.999 |
|  |  |  |  |  |  |  |

**Supplementary Figure 2**. Time to study outcomes according to oral anticoagulation status in the unweighted population.

**Supplementary Table 3**. Comparison of baseline characteristics between patients taking and those not taking drugs interacting with oral anticoagulants. Standardized mean differences are reported before and after inverse probability weighing.

|  | **No interacting drugs**  **(n=1180)** | **Interacting drugs**  **(n=469)** | **SMD (unweighted)** | **SMD (weighted)** |
| --- | --- | --- | --- | --- |
| Age (median-[IQR]) |  |  |  |  |
| Female, n (%) | 583 (49.4) | 206 (43.9) | 0.110 | 0.035 |
| Ethnicity, n (%) |  |  | 0.276 | 0.122 |
| Caucasian | 912 (77.3) | 391 (83.4) |  |  |
| Hispanic white | 73 (6.2) | 40 (8.5) |  |  |
| Black | 18 (1.5) | 6 (1.3) |  |  |
| Asian | 7 (0.6) | 2 (0.4) |  |  |
| Other | 170 (14.4) | 30 (6.4) |  |  |
| Hospitalized |  |  | 0.079 | 0.110 |
| Stroke unit | 1078 (94.6) | 426 (93.2) |  |  |
| Intensive care unit | 30 (2.6) | 12 (2.6) |  |  |
| Other hospital unit | 31 (2.7) | 19 (4.2) |  |  |
| Risk factors (n, %): |  |  |  |  |
| Smoking | 126 (10.7) | 28 (6.0) | 0.171 | 0.115 |
| Hypertension | 922 (78.1) | 416 (88.7) | 0.187 | 0.026 |
| Dyslipidemia | 560 (47.5) | 285 (60.8) | 0.270 | 0.021 |
| Diabetes | 302 (25.6) | 141 (30.1) | 0.100 | 0.140 |
| Prior Myocardial Infarction | 227 (19.2) | 139 (29.6) | 0.244 | 0.020 |
| Prior Stroke/TIA | 281 (23.8) | 129 (27.5) | 0.085 | 0.034 |
| Prior ICH | 20 (1.7) | 7 (1.5) | 0.016 | 0.052 |
| Chronic heart failure | 168 (14.2) | 109 (23.2) | 0.232 | 0.037 |
| Chronic liver failure | 4 (0.3) | 3 (0.6) | 0.043 | 0.048 |
| Chronic kidney failure | 158 (13.4) | 110 (23.5) | 0.262 | 0.011 |
| Peripheral artery disease | 44 (3.7) | 36 (7.7) | 0.171 | 0.042 |
| Valvular heart disease |  |  | 0.138 | 0.172 |
| Mitral stenosis | 24 (2.0) | 9 (1.9) |  |  |
| Aortic stenosis | 16 (1.4) | 15 (3.2) |  |  |
| Mitral insufficiency | 31 (2.6) | 15 (3.2) |  |  |
| Aortic insufficiency | 6 (0.5) | 1 (0.2) |  |  |
| Multiple | 11 (0.9) | 4 (0.9) |  |  |
| Mechanical valve replacement | 60 (5.1) | 34 (7.2) | 0.090 | 0.118 |
| Biological valve replacement | 50 (4.2) | 31 (6.6) | 0.105 | 0.088 |
| Pacemaker | 123 (10.4) | 88 (18.8) | 0.238 | 0.104 |
| Atrial fibrillation type |  |  | 0.329 | 0.085 |
| Paroxysmal | 219 (18.6) | 116 (24.7) |  |  |
| Persistent | 167 (14.2) | 33 (7.0) |  |  |
| Long-standing persistent | 53 (4.5) | 23 (4.9) |  |  |
| Permanent | 585 (49.6) | 262 (55.9) |  |  |
| Not classified | 156 (13.2) | 35 (7.5) |  |  |
| Anticoagulation type |  |  | 0.075 | 0.026 |
| VKA | 257 (21.8) | 117 (24.9) |  |  |
| DOAC | 923 (78.2) | 352 (75.1) |  |  |
| Minor stroke | 347 (29.4) | 145 (30.9) | 0.033 | 0.074 |
| Minor to moderate stroke | 578 (49.0) | 223 (47.5) | 0.029 | 0.013 |
| NIHSS baseline (median) |  |  |  |  |
| Large vessel occlusion | 648 (54.9) | 264 (56.3) | 0.069 | 0.039 |
| IVT | 101 (8.6) | 38 (8.1) | 0.017 | 0.038 |
| EVT | 508 (43.1) | 224 (47.8) | 0.095 | 0.084 |
|  |  |  |  |  |

**Supplementary Figure 3**. Balance plot of covariates before and after inverse probability weighing.
